# Preoperative Hemostatic Management for Refractory Abnormal Uterine Bleeding in Patients With von Willebrand Disease: A Case Report

**DOI:** 10.1155/crog/5078814

**Published:** 2026-02-25

**Authors:** Rabbania Hiksas, Lisa Novianti, Intan Indah Permatasari, Thalia Amila Elsiyana, Aisyah Retno Puspawardani, Achmad Kemal Harzif

**Affiliations:** ^1^ Department of Obstetrics and Gynecology, Dr. Cipto Mangunkusumo General Hospital, Faculty of Medicine, Universitas Indonesia, Jakarta, Indonesia, ui.ac.id; ^2^ Human Reproduction, Infertility, and Family Planning Cluster, Indonesia Reproductive Medicine Research and Training Center, Faculty of Medicine, Universitas Indonesia, Jakarta, Indonesia, ui.ac.id; ^3^ Reproductive Immunoendocrinology Division, Department of Obstetrics and Gynecology, Dr. Cipto Mangunkusumo General Hospital, Faculty of Medicine, Universitas Indonesia, Jakarta, Indonesia, ui.ac.id

**Keywords:** abnormal uterine bleeding, coagulopathy, perioperative management, von Willebrand disease

## Abstract

**Introduction:**

von Willebrand disease (VWD) is the most frequent inherited bleeding disorder in women, characterized by quantitative or qualitative deficiency in von Willebrand factor (VWF). This deficiency in functional VWF has a dual effect on hemostasis, resulting in impaired coagulation; thus, it may cause unstoppable abnormal uterine bleeding (AUB).

**Case Report:**

A 44‐year‐old female complained of AUB for 4 years before admission. She was diagnosed with adenomyosis and had been taking dienogest 1 × 2 mg; however, the bleeding still continued. She was hospitalized due to her heavy bleeding, with her lowest hemoglobin level (7.0 g/dL) and received recurrent blood transfusion. As coagulation disorder was suspected, the laboratory result revealed a low level of Factor VIII (52.0%) and Factor von Willebrand (37.0%), suggestive of Type 2 VWD. She also had generalized anxiety disorder related to her refractory AUB. She then underwent laparoscopy total hysterectomy, right salpingo–oophorectomy, and left salpingectomy for source control. Preoperatively, 2 units of cryoprecipitate and intranasal desmopressin (DDAVP) 300 mcg were given. The medication continued up to 7 days after surgery, with no massive bleeding reported afterwards.

**Conclusion:**

Coagulopathies, including VWD, should always be suspected in patients with refractory AUB. Preoperative medication such as cryoprecipitate products and DDAVP could be given to provide an immediate hemostatic response in preventing excessive bleeding during surgery. Laparoscopy is considered the best surgical approach to ensure less blood loss and lower risk of hemorrhage. A multidisciplinary approach is essential to ensure both effective hemostatic control and comprehensive patient care.

## 1. Introduction

von Willebrand disease (VWD) is the most common inherited bleeding disorder in women, resulting from a deficiency or dysfunction of von Willebrand factor (VWF), a glycoprotein essential for platelet adhesion and stabilization of Factor VIII (FVIII) [1]. VWD can present with a range of bleeding symptoms, including easy bruising, epistaxis, and prolonged bleeding after surgery or trauma. In women, abnormal uterine bleeding (AUB) is a frequent but often under‐recognized manifestation, significantly impacting quality of life and increasing the risk of anemia [2]. Despite its prevalence, VWD‐related AUB remains a diagnostic challenge due to its varied clinical presentations and overlap with other gynecological conditions. AUB in patients with VWD is often misdiagnosed as dysfunctional uterine bleeding or attributed to hormonal imbalances, leading to delayed or inappropriate treatment. The subtle and chronic nature of bleeding symptoms may result in a late diagnosis. Many women undergo multiple failed treatments with hormonal therapy or even unnecessary surgical interventions before hematologic evaluation is considered [2]. Therefore, early recognition and appropriate laboratory testing, including von Willebrand factor antigen (VWF:Ag) and FVIII levels, are crucial for accurate diagnosis.

This case report highlights a patient with VWD presenting with persistent AUB, initially misdiagnosed as a gynecologic disorder. By reviewing the diagnostic pitfalls and exploring effective management strategies, including hormonal therapy, antifibrinolytics, and desmopressin (DDAVP), we are aimed at emphasizing the importance of a multidisciplinary approach. Proper recognition and individualized treatment can prevent complications, improve patient outcomes, and reduce the burden of unnecessary interventions in women with underlying bleeding disorders.

## 2. Case Presentation

**Figure 1:**
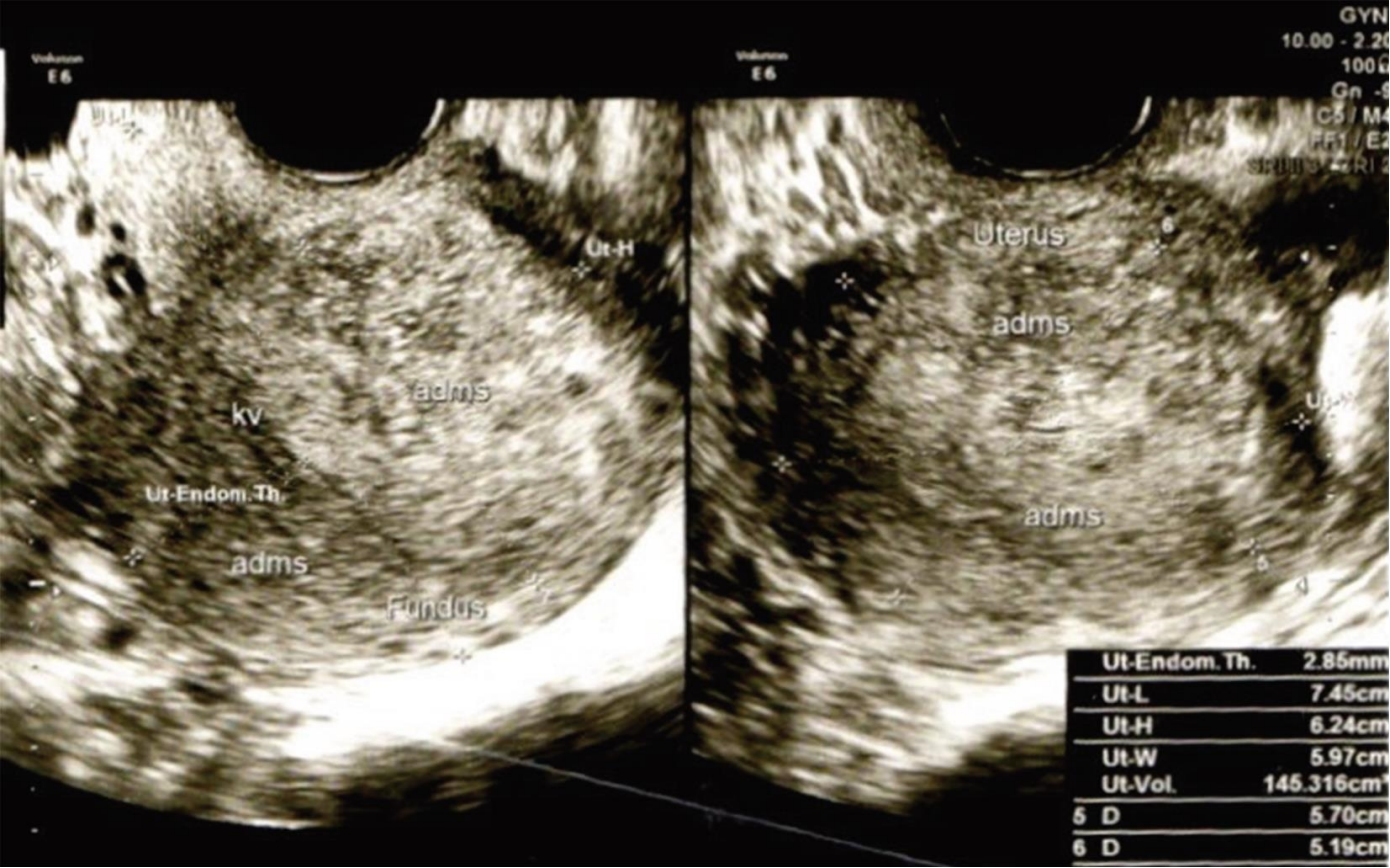
Imaging findings. Ultrasound revealed enlargement of globular shaped uterus, retroflexion with volume 145 cm^3^, not suppressing the uterine cavity EL 2.84 mm, normal cervix portio and endocervix, with no seen mass on both ovaries.

A 44‐year‐old female presented with AUB that had persisted for 4 years. Her menstrual periods were consistently heavy, requiring up to 4–5 pad changes per day, and often lasted up to 10 days. Pelvic ultrasound revealed a globular uterus with a heterogeneous hyper–hypoechoic lesion with irregular borders in the posterior corpus, measuring 57 × 51 × 48 mm, consistent with diffuse adenomyosis (Figure 1). She had been treated with dienogest 2 mg daily, but her bleeding persisted. On several occasions, she required hospitalization due to heavy bleeding, with the lowest recorded hemoglobin of 7.0 g/dL, necessitating repeated blood transfusions.

There was no family history of bleeding disorders. However, detailed history‐taking revealed frequent epistaxis and easy bruising since early childhood. Menarche was followed by progressively heavier and prolonged menses, sometimes lasting up to 3 months during adolescence. In 2012, she delivered her first child by cesarean section; although intraoperative bleeding was not excessive, she developed extensive postoperative bruising that resolved slowly, and her postpartum bleeding remained heavy for up to 3 months. She also reported occasional unexplained bruising on the extremities and prolonged bleeding from minor cuts but did not seek medical evaluation due to perceiving these symptoms as insignificant. During her most recent hospitalization, she required multiple blood transfusions. Based on her bleeding history, the patient′s *International Society on Thrombosis and Haemostasis–Scientific and Standardization Committee Bleeding Assessment Tool (ISTH-SSC BAT)* score was 6, raising suspicion of an underlying bleeding disorder.

Given concerns for a coagulopathy, a hematology consultation was obtained. Laboratory evaluation as shown in Table 1 demonstrated a Factor VIII level of 52% and VWF:Ag of 37%, both mildly reduced. The VWF activity (GPIb binding assay) was 38%, with a VWF activity to VWF:Ag ratio of 1.7, significantly above the < 0.7 threshold typically associated with qualitative VWF defects. Platelet function testing by light transmission aggregometry (LTA) showed normal aggregation responses to ADP at multiple concentrations. These findings demonstrated a proportional reduction in VWF antigen and activity, a laboratory pattern compatible with a quantitative VWF deficiency, most consistent with VWD Type 1. Essential qualitative assays such as multimer analysis, von Willebrand Factor collagen‐binding assay (VWF:CB), low‐dose ristocetin‐induced platelet aggregation (RIPA), or genetic testing were not performed.

**Table 1:** Laboratory parameters suggestive of von Willebrand disease.

	Result	Unit	Normal range
von Willebrand factor	37	%	—
Factor VIII	52	%	60–150
APTT patient	32.1	Second	
APTT control	29.9	Second	
ADP 1 *μ*M	4.7	%	3.0–15.0
ADP 2.5 *μ*M	6.9	%	5.0–35.0
ADP 5 *μ*M	79.2	%	25.0–68.0
ADP 10 *μ*M	77.7	%	49.0–84.0
VWF Activity (GPIb binding assay)	38	%	44–141% (for her O blood type)
Activity: antigen ratio	1.7	—	—

Serial platelet counts remained within the mildly reduced to low–normal range—144,000, 119,000, 141,000, and 168,000/*μ*L—with no evidence of severe thrombocytopenia.

Iron studies demonstrated low ferritin (18.42 ng/mL) with normal transferrin saturation, serum iron, and total iron‐binding capacity, consistent with early iron deficiency without overt iron‐deficiency anemia. She was started on oral iron supplementation (Maltofer) and instructed to continue therapy to restore iron stores.

The patient reported increasing anxiety over the previous 6 months, which she associated with the recurrence of heavy bleeding. She attributed fatigue, presyncope, and emotional distress to longstanding anemia and repeated hospital visits. She was evaluated by a psychiatrist and diagnosed with generalized anxiety disorder, for which she was started on quetiapine IR 50 mg once daily and alprazolam 0.5 mg once daily.

Based on the laboratory findings compatible with a quantitative VWF deficiency, she received DDAVP at a dose of 0.3 *μ*g/kg intranasally (20 *μ*g single dose), followed by oral DDAVP 0.4 mg twice daily, resulting in significant improvement in her bleeding symptoms. Given her refractory AUB despite medical therapy, she was scheduled for an elective laparoscopic hysterectomy with right salpingo–oophorectomy and left salpingectomy.

As part of preoperative hemostatic optimization, she received 2 units of cryoprecipitate, intranasal DDAVP 300 *μ*g, and intravenous tranexamic acid at 10 mg/kg (~630 mg) prior to surgery. Tranexamic acid was continued postoperatively at 25 mg/kg orally every 8 h for 5–7 days. These measures were instituted to minimize perioperative bleeding risk.

Laparoscopy revealed an enlarged uterus consistent with adenomyosis (Figure 2a). A total hysterectomy, right salpingo–oophorectomy, and left salpingectomy were performed uneventfully. The surgical specimen is shown in Figure 2b.

Figure 2(a) Laparoscopic procedure and (b) gross specimen.

**Figure:**
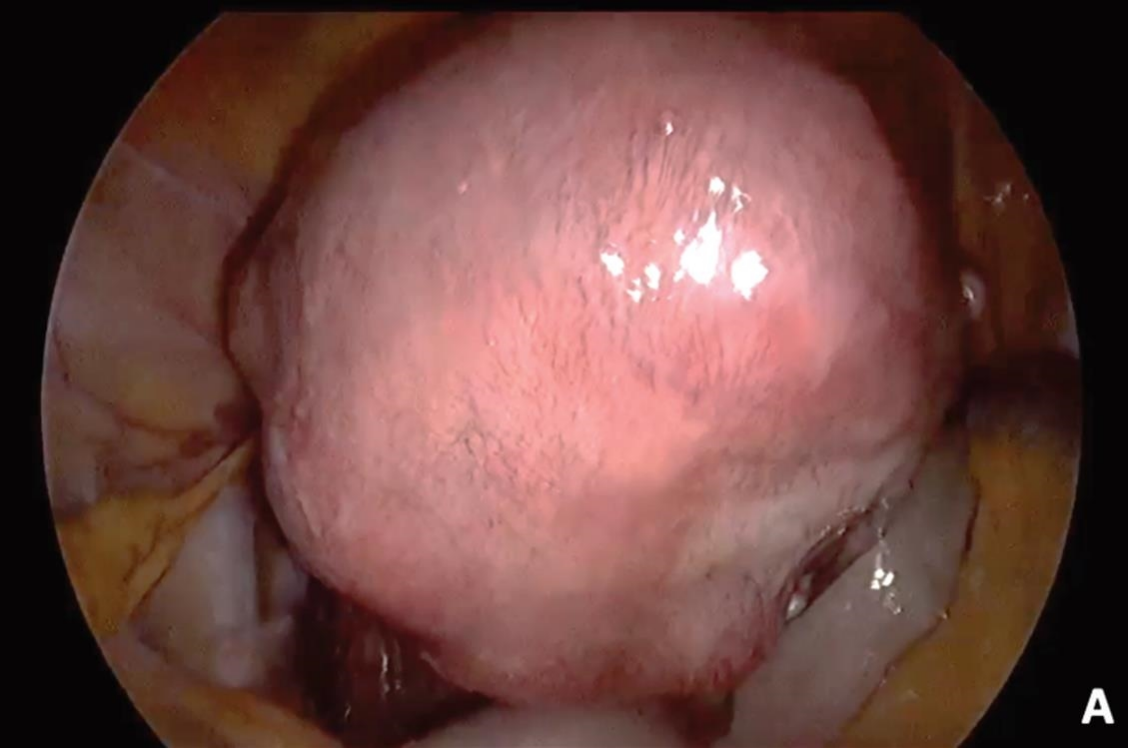
(a)

**Figure:**
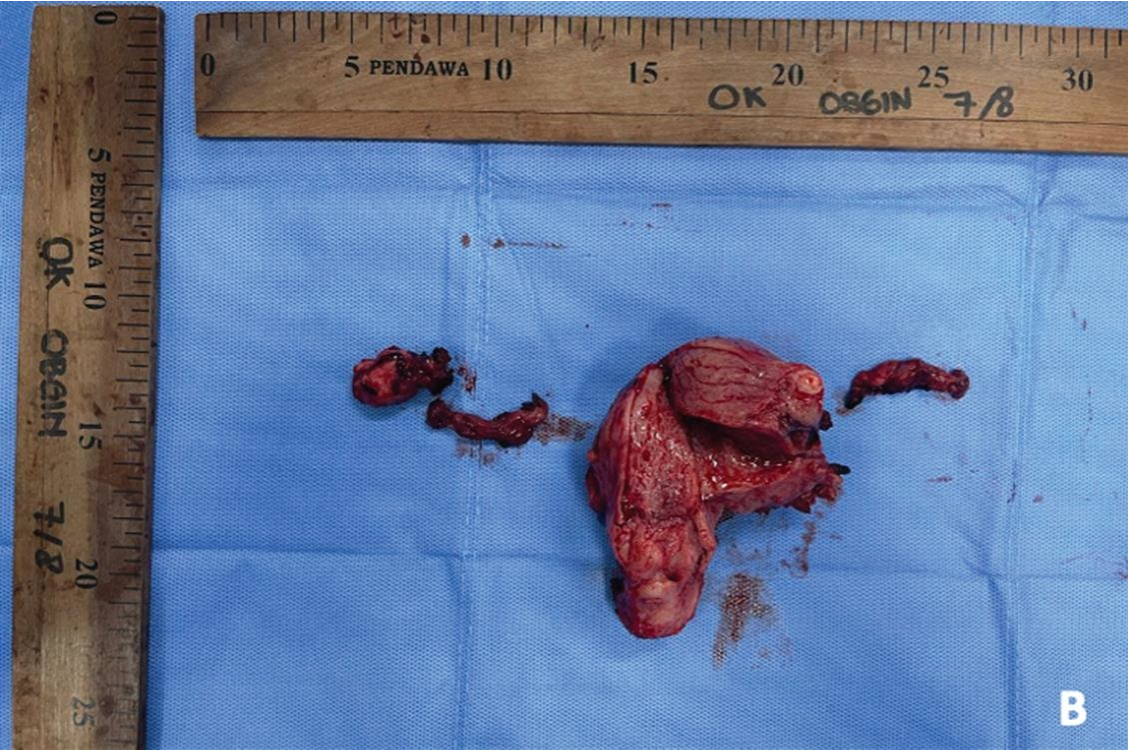
(b)

Postoperative recovery was favorable. The patient remained hemodynamically stable without episodes of massive bleeding. Hemostatic support with DDAVP and antifibrinolytic therapy was continued for 7 days postoperatively.

## 3. Discussion

VWD is the most common inherited bleeding disorder and results from quantitative or qualitative abnormalities in VWF, a key protein involved in platelet adhesion and FVIII stabilization during hemostasis [3]. Defective VWF leads to mucocutaneous bleeding manifestations such as epistaxis, easy bruising, and heavy menstrual bleeding which in women frequently appears as AUB [4]. Because AUB is often attributed solely to gynecologic conditions, inherited bleeding disorders such as VWD may remain undiagnosed for many years, allowing chronic bleeding to contribute to significant morbidity including anemia and impaired quality of life [5]. In this case, the clinical history of lifelong bleeding tendencies—particularly heavy menstrual bleeding from adolescence—combined with recurrent symptomatic anemia requiring transfusions, provided a strong indication of an underlying bleeding disorder. The elevated ISTH‐BAT score of 6 further supported this suspicion as validated in the diagnostic workup of inherited bleeding conditions [6].

Laboratory testing revealed reduced VWF:Ag (37%), VWF activity (38%), and mildly decreased FVIII levels (52%) with a VWF activity to VWF:Ag ratio of 1.7. A ratio above 0.7 reflects a proportional decrease in both activity and antigen, which is characteristic of a quantitative VWF deficiency and supports a diagnosis compatible with VWD Type 1 [3, 7]. Platelet function testing using ADP demonstrated normal aggregation, consistent with preserved qualitative platelet function in Type 1. Although, advanced assays such as VWF multimer analysis, VWF collagen binding, low‐dose RIPA, and genetic testing would further refine subtype classification, these are not universally available in clinical practice and are not strictly required when clinical phenotype and first‐line laboratory evaluation already align with a diagnosis of VWD Type 1 [3]. The absence of severe thrombocytopenia also supports this classification [7]. Although definitive VWD subclassification ideally requires multimer analysis, VWF collagen‐binding assays, or genetic testing, the proportional reduction of VWF antigen and activity with an activity − to − antigen ratio > 0.7 strongly supports a quantitative VWF deficiency pattern in this patient.

Chronic heavy menstrual bleeding frequently causes iron deficiency even prior to the onset of overt anemia [8]. In this patient, ferritin was low despite normal iron indices, indicating early iron depletion requiring ongoing supplementation. In addition to hematologic impacts, the psychological burden of recurrent uncontrolled bleeding is increasingly recognized. Women with AUB often experience anxiety, fear of hospitalization, reduced productivity, and diminished overall well‐being, particularly when the bleeding disorder remains untreated [9, 10]. The patient′s development of generalized anxiety disorder illustrates the need for integrated multidisciplinary care to address both physical and psychological consequences.

Management of VWD Type 1 focuses on therapies that enhance endogenous VWF release or stabilize clots. DDAVP is considered first‐line for Type 1 VWD due to its proven ability to increase plasma VWF and FVIII concentrations [3]. The patient had a previously documented clinical response to DDAVP and the 2021 guidelines jointly issued by the American Society of Hematology, the International Society on Thrombosis and Haemostasis, the National Hemophilia Foundation, and the World Federation of Hemophilia state that when response is known, a repeat DDAVP test is not required prior to surgery [3]. Tranexamic acid also plays a critical role by inhibiting fibrinolysis and thereby reducing perioperative bleeding risk, particularly in procedures involving mucosal surfaces [3, 11]. Both therapies were appropriately implemented before and after surgery in this case. Because VWF concentrates are not widely accessible in all healthcare settings, cryoprecipitate remains a practical alternative and was used preoperatively to raise VWF, FVIII, and fibrinogen levels consistent with local resource availability [3].

Definitive surgical management with laparoscopic hysterectomy was indicated due to persistent AUB despite hormonal therapy and significant impact on health status. Minimally invasive surgery is preferred in patients with bleeding disorders because it reduces operative blood loss, postoperative pain, and wound complications, ultimately improving recovery outcomes [12]. Preservation of ovarian tissue was also appropriate to prevent premature ovarian insufficiency and its long‐term risks including osteoporosis, cardiovascular complications, and adverse metabolic effects [13]. The favorable postoperative course—with no major bleeding events—demonstrates the effectiveness of a structured, guideline‐based perioperative hemostatic plan.

This case underscores the importance of early recognition of bleeding disorders in women with longstanding heavy menstrual bleeding. Implementing appropriate diagnostic evaluation and timely initiation of VWD‐specific therapy can significantly reduce morbidity, improve surgical outcomes, and enhance quality of life. Moreover, multidisciplinary collaboration among gynecology, hematology, and psychiatry played a key role in achieving successful management for this patient. Overall, comprehensive care—including DDAVP, tranexamic acid, iron therapy, and minimally invasive definitive surgery—resulted in complete resolution of symptoms and effective control of bleeding in a patient with VWD Type 1.

## 4. Conclusion

This case highlights the importance of evaluating underlying bleeding disorders, such as VWD, in women with persistent heavy menstrual bleeding that does not improve with standard therapy. Early recognition supported timely and appropriate hemostatic optimization prior to surgery. The combination of desmopressin, antifibrinolytics, iron supplementation, and cryoprecipitate, followed by minimally invasive hysterectomy, resulted in safe bleeding control and definitive symptom resolution. Additionally, psychological support played a vital role in addressing the emotional burden associated with recurrent bleeding and anemia. A multidisciplinary approach is essential to improve clinical outcomes and overall quality of life in patients with refractory AUB due to VWD.

## Funding

No funding was received for this manuscript.

## Ethics Statement

This case report has obtained approval from the Ethics and Research Committee of the Faculty of Medicine at Universitas Indonesia.

## Consent

The participant in this study gave informed consent after being fully informed about the study′s objectives, procedures, potential risks, and benefits. The participant willingly agreed to take part and has provided permission to publish the case.

## Conflicts of Interest

The authors declare no conflicts of interest.

## Data Availability

The datasets used and/or analyzed during the current study are available from the corresponding author on reasonable request.
